# Retail Chicken Carcasses as a Reservoir of Multidrug-Resistant *Salmonella*

**DOI:** 10.1089/mdr.2021.0414

**Published:** 2022-07-13

**Authors:** Sara H. Al-Hadidi, Hassan Al mana, Salam Ziad Almoghrabi, Tahra El-Obeid, Walid Q. AlAli, Nahla O. Eltai

**Affiliations:** ^1^ Biomedical Research Center, Qatar University, Doha, Qatar.; ^2^ Department of Health Nutrition, College of Health Sciences, Qatar University, Doha, Qatar.; ^3^ Department of Epidemiology and Biostatistics, Faculty of Public Health, Kuwait University, Safat, Kuwait.

**Keywords:** *Salmonella*, retail chicken, foodborne, AMR

## Abstract

is a major cause of foodborne disease outbreaks worldwide, mainly through poultry. Recently, there has been an increase in multidrug-resistant (MDR) *Salmonella* infections globally. The increased drug resistance results in increased costs and poorer health outcomes due to unavailability or delayed treatment. This study aims to determine the prevalence of *Salmonella* in retail raw chicken meat and identify their antimicrobial resistance profiles. A total of 270 retail raw chicken carcasses (local and imported) were collected from three hypermarket chains in Qatar between November 2017 and April 2018. Thirty carcasses were contaminated with *Salmonella* (11.11%). The prevalence of *Salmonella* in locally produced chicken was higher than in imported chicken (OR = 2.56, 95% CI: 1.18–5.53, *p* = 0.016). No significant differences were found between the prevalence and storage temperature or hypermarket chain. The highest resistance rates in the isolates were reported to tetracycline (73.7%) followed by nitrofurantoin (53.3%), ampicillin (50%), amoxicillin-clavulanic acid, ceftriaxone (26.7%), and ciprofloxacin (23.3%). Eight isolates were potential extended-spectrum β-lactamase-producers, all in imported frozen chicken (*p* < 0.0001). Additionally, 43.3% of the isolates were MDR and associated with frozen chicken (OR = 16.88, 95% CI: 2.55–111.47, *p* = 0.002). The findings indicate that while the prevalence of *Salmonella* in retail chicken in Qatar is moderate, a large proportion of them are MDR.

## Introduction

*Salmonella (S)* is one of the most common zoonotic foodborne pathogens that cause outbreaks and sporadic cases of gastroenteritis in humans throughout the world.^1^ The genus consists of two species, *S. bongori* and *S. enterica*. The latter is divided into seven subspecies (*arizonae*, *diarizonae*, *enterica*, *houtenae*, *indica*, *salamae*, and subspecies VII).^2^ The majority of salmonellosis cases are due to *S. enterica* subsp. *enterica*. It contains over 2500 serovars (based on serological typing of the O-and H-antigens), most of which cause diseases in humans.^2–5^

The main reservoirs for *Salmonella* are the gastrointestinal tracts of livestock animals, which may lead to the contamination of food products.^6^ As such, salmonellosis is typically associated with the handling and consuming food of animal origin. Salmonellosis is among the most common foodborne infections worldwide, constituting a significant healthcare and economic burden.^7^ Chickens are thought to be the leading risk factor for human salmonellosis, as they are frequently asymptomatically colonized by non-typhoidal *Salmonella* (*i.e.,* serovars other than Typhi and Paratyphi).^8^

While salmonellosis is typically self-limiting, *Salmonella* can cause invasive disease, which requires the use of antibiotics. Resistance to ampicillin, chloramphenicol, and sulfamethoxazole-trimethoprim has been reported in *Salmonella* for many years, which shifted treatment toward fluoroquinolones and extended-spectrum cephalosporins.^9^ However, recent years have seen an increase in multidrug-resistant (MDR) *Salmonella* infections, further increasing the significance of this pathogen and its healthcare burden.^10^

*Salmonella* is not naturally transformable, meaning that it can only develop antimicrobial resistance (AMR) through mutations or acquisition from both within the genus and with other genera.^11–13^ Animal housing conditions and antimicrobial use in farms are thought to facilitate AMR dissemination between *Salmonella* species and the acquisition of genes from other related species.^14^ Additionally, the conditions in poultry processing facilities can promote the growth of *Salmonella* biofilms on both biotic and abiotic surfaces, which in turn may promote AMR acquisition.^15^ Evidence indicates that poultry act as a reservoir for antimicrobial-resistant *Salmonella* and links to human antimicrobial-resistant salmonellosis.^6^

Antimicrobial-resistant bacteria, including *Salmonella*, can be transmitted from animals to humans or vice-versa by direct contact or indirectly through animal products and the environment.^16^ The interconnectedness of humans, animals, and the environment in AMR transmission increases the need to adopt a one health approach. Food products represent a link between humans and animals. Thus, they are a potential source of transmission and bioaccumulation.^17^ Moreover, a potential *Salmonella* transmission link between poultry and humans has been previously reported.^18–21^ MDR *Escherichia coli* have been reported previously in Qatar in both humans and animals, including chicken and sheep.^22–25^ To complete the picture, studies on AMR in food are necessary. In this study, we investigate the prevalence of *Salmonella* in retail chicken and report its AMR profile.

## Materials and Methods

### Sample collection

The retail chicken carcasses used in this study were described in our previous study.^24^ Briefly, a total of 270 chicken carcasses were used in this study were collected from two branches of each of three major hypermarket chains (designated A, B, and C) in Doha and Al-Rayyan municipalities in Qatar from November 2017 to April 2018 using a stratified random sampling method (Table 1). The retail chicken samples were transferred in cooled boxes (4°C–8°C) to the Qatar University Biomedical Research Center (Doha, Qatar) laboratories. Upon arrival, the samples were stored at 4°C and processed within 24 hrs. Information on the store name, location, storage temperature (chilled or frozen), collection date, source (local or imported), producer, and sell-by date (or expiration date) were recorded. Research approval to process samples was obtained from Qatar University's Institutional Biohazard Committee under approval number QU (QU-IBC-2018/034).

**Table 1.** Number and Location of the Chicken Meat Samples Collected from Hypermarket Stores by Storage Temperature, Chicken Source, and Municipality in Qatar (*n* = 270)

Store	Storage temperature	Source^a^	Number of samples (Doha)	Number of samples (AL-Rayyan)
Hypermarket A	Chilled	Local	15	15
	Chilled	Imported	15	15
	Frozen	Imported	15	15
Hypermarket B	Chilled	Local	15	15
	Chilled	Imported	15	15
	Frozen	Imported	15	15
Hypermarket C	Chilled	Local	15	15
	Chilled	Imported	15	15
	Frozen	Imported	15	15
Total			135	135

^a^ There were no frozen local chicken available in the hypermarkets. All local chicken sold at the time of the study was chilled.

### *Salmonella* isolation and identification

**FIG. 1.**
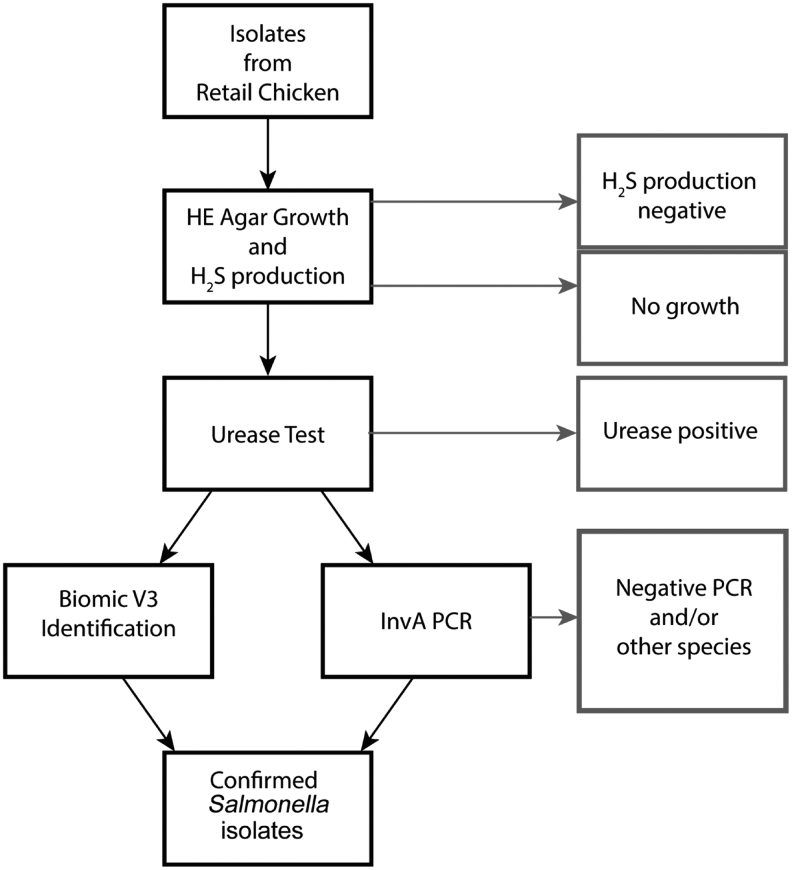
Flowchart of the *Salmonella* isolation and identification process. The flow chart describes the sequential process that was used to determine whether a chicken carcass was contaminated with *Salmonella*. The *red boxes* on the *right* detail the exclusion criteria (*i.e.,* sample is not contaminated by *Salmonella*) at each step of the process. First, the chicken carcasses were homogenized and mixed with SCB to enrich for *Salmonella*. Second, 20 μL of the SCB broth-homogenate mixture was streaked on HE agar to select for *Salmonella* and *Shigella*. HE agar differentiates between the two species through H_2_S production in *Salmonella*, which results in *black* colonies. The samples that did not have growth on HE agar or had growth without H_2_S production were determined not to be contaminated with *Salmonella* and excluded from subsequent steps. Third, a urease test was performed on the H_2_S-producing isolates. *Salmonella* is urease negative, as such, the urease positive were excluded from the next steps, and the chicken carcasses they came from were determined not to be contaminated with *Salmonella*. The last two steps in the process were PCR for the *invA* gene (A conserved gene in *Salmonella*) and biochemical identification with the Biomic V3 platform. The process resulted in identifying 30 retail chicken carcasses that were positive for Salmonella. SCB, selenite cystine broth; HE, Hektoen Enteric.

The *Salmonella* isolation and identification process is summarized in Fig. 1. Each chicken carcass was soaked in 250 mL of sterilized buffered peptone water (HiMedia, Mumbai, India), vigorously shaken for 1 min, and incubated at 37°C for 30 min at 200 rpm. Then, 50 mL of the rinsate was inoculated into 50 mL of sterile selenite cystine broth (HiMedia, Mumbai, India) for selective enrichment of *Salmonella*. The rinsate-broth mixture was incubated for 24 h at 37°C in a shaking incubator at 100 rpm. A loop-full (20 μL) of broth culture was then streaked onto Hektoen Enteric (HE) Agar (HiMedia) and incubated at 37°C for 16–20 h. HE agar is a selective and differential medium that selects *Salmonella* and *Shigella*. *Salmonella* is detected by the presence of black colonies due to the production of H2S.

Three separate black colonies were picked from each *Salmonella*-positive plate, subcultured onto nutrient agar (HiMedia), and incubated at 37°C for 16–20 h. The urease test was then performed to confirm the identity. For each nutrient agar plate, two to three colonies were inoculated into a Urea broth tube, such that each tube corresponds to a nutrient agar plate. The broth tubes were then incubated at 35°C for 18–24 h. *Salmonella* is urease-negative; thus, no change in broth color would be observed. The identities of the urease negative isolates were then further confirmed to be *Salmonella* through polymerase chain reaction (PCR) of the conserved invasion A (*invA*) gene and biochemically using the Biomic V3 System (Giles Scientific) with the BBL CRYSTAL™ Enteric/Nonfermenter (E/NF) id KIT (BD).

For PCR, total DNA was extracted from the urease negative isolates using the QIAamp^®^ UCP Pathogen mini kit (Qiagen, Germany) following the manufacturer's protocol. The PCR for *invA* was performed using the primers and procedure described by Naik *et al*.^26^ The PCR was performed on a Biometra TAdvanced thermocycler (Analyticjena, Germany), and the results were visualized through agarose gel electrophoresis.

It should be noted that the two methods used to confirm the *Salmonella* identity do not differentiate the serovars.

### Antibiotic susceptibility testing

All *Salmonella* isolates obtained from retail chicken carcasses were tested for their susceptibility to a relevant panel of antibiotics. A single pure *Salmonella* colony from each nutrient agar plate was inoculated into a phosphate buffer solution (Atom Scientific) to achieve a 0.5 McFarland inoculum, measured by DensiCHEK PLUS (bioMérieux). The suspension was then swabbed onto a Mueller–Hinton agar plate (HiMedia) and allowed to dry completely. Next, antibiotic-impregnated discs (Liofilchem^®^, Roseto degli Abruzzi) were applied to the agar surface (up to 6 per plate) and incubated at 37°C for 24 hrs. The zone of inhibition was measured in mm and interpreted as per the Clinical and Laboratory Standards Institute (CLSI) guidelines.^27^

The antibiotic susceptibility panel included 13 disks: Ampicillin (AMP, 10 μg), Amoxicillin/Clavulanic acid (AMC, 20/10 μg), Tetracycline (TET, 30 μg), Piperacillin/Tazobactam (TZP, 100/10 μg), Ciprofloxacin (CIP, 5 μg), Trimethoprim/Sulfamethoxazole (SXT, 1.25/23.75 μg), Ceftriaxone (CRO, 30 μg), Cefepime (FEP, 30 μg), Fosfomycin (FOS, 200 μg), Nitrofurantoin (F, 300 μg), Ertapenem (ETP, 10 μg), and Meropenem (MRP, 10 μg). Colistin susceptibility was measured using E-test (Liofilchem) following the manufacturer's instructions to avoid the possible false-negative results with Colistin resistance. *E. coli* ATCC 25922 was used as a negative control. Non-susceptible isolates to any third-generation cephalosporins were considered potential extended-spectrum β-lactamase (ESBL) producers.

### Data analysis

Statistical analysis was performed using R version 4.1.0.^28^ For statistical analysis, all intermediate resistances were recoded as susceptible. The *χ*^2^-test of independence was used to determine whether significant associations existed between the presence of *Salmonella* or phenotypic antibiotic resistances and storage temperature (chilled or frozen), chicken source (local or imported), hypermarket (A, B, or C), or municipality (Doha or Al-Rayyan). The Binomial test was used to determine whether the difference between the proportions of sensitive and resistant isolates per antibiotic is significant. The Goodman–Kurksal tau was used to measure pair-wise associations between the resistance phenotypes. A *p*-value <0.05 was considered statistically significant. All plots were generated using the ggplot2 version 3.3.3 and ggpubr version 0.4.0 packages in R.^29,30^

## Results

### Prevalence of *Salmonella* in retail chicken

Thirty retail chicken carcasses were positive for *Salmonella* as identified by *invA* PCR and Biomic V3 identification (Supplementary Fig. S1 and Supplementary Table S1). The total prevalence of *Salmonella* in chicken carcasses was 11.11%. Hypermarket A had the highest prevalence with 13.3% of the samples containing *Salmonella*, followed by hypermarkets B and C with 11.1% and 8.89%, respectively (Table 2). There was a significant difference between local and imported chicken, with higher odds in local chicken (OR = 2.56, 95% CI: 1.18–5.53, *p* = 0.016). All local chicken was chilled; however, no significant difference was observed when the storage temperatures were compared (10.6% vs. 12.2%; *p* = 0.84). The origins of the imported chicken samples included in the study were from Brazil for the frozen chicken and France, The Netherlands, Pakistan, and Turkey for the chilled samples. No *Salmonella* was isolated from chilled imported chicken from hypermarket B.

**Table 2.** The Prevalence of *Salmonella* in Three Hypermarket Chains (A–C) in Qatar

Chicken type	Number of samples positive for Salmonella (%)^a^			
	A	B	C	Total
Local chilled	5 (5.56)	7 (7.78)	4 (4.44)	16 (17.8)
Imported chilled	1 (1.11)	0	2 (2.22)	3 (3.33)
Imported frozen	6 (6.67)	3 (3.33)	2 (2.22)	11 (12.2)
Total	12 (13.3)	10 (11.1)	8 (8.89)	30

^a^ Thirty specimens were collected from each of the three types of chicken from each hypermarket (15 from each municipality), for a total of 270 chicken specimen.

### Phenotypic resistance profile of *Salmonella* in retail chicken

**FIG. 2.**
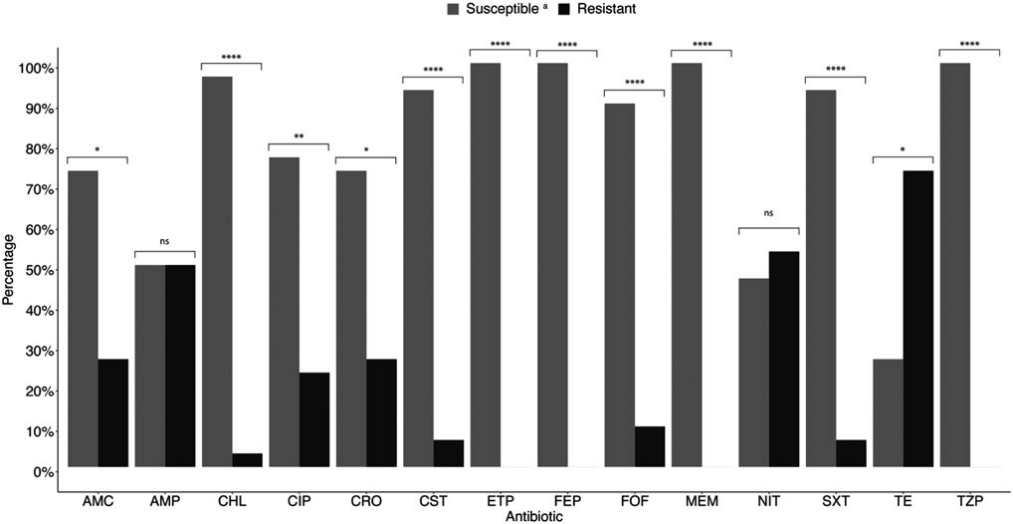
The Antibiotic resistance profiles in the *Salmonella* isolated from retail chicken carcasses in Qatar (*n* = 30). ^a^Isolates were classified as susceptible if they were sensitive (S) or intermediate (I) with *in vitro* antibiotic susceptibility testing. A binomial test was performed to determine whether the difference in proportions between susceptible and resistant isolates is significant. ns: not significant (*p* > 0.05), *p ≤ 0.05, **p ≤ 0.01, ****p ≤ 0.0001. AMC, amoxicillin-clavulanic acid; AMP, ampicillin; CHL, chloramphenicol; CIP, ciprofloxacin; CRO, ceftriaxone; CST, colistin; ETP, ertapenem; FEP, Cefepime; FOF, Fosfomycin; MEM, meropenem; NIT, nitrofurantoin; SXT, sulfamethoxazole-trimethoprim; TE, tetracycline; TZP, piperacillin-tazobactam.

The antibiotic resistance profiles of the *Salmonella* isolated from the 30 retail chicken carcasses are summarized in Fig. 2 and Table 3. All the *Salmonella* isolates were susceptible to carbapenems, the 4th-generation cephalosporin cefepime, and the piperacillin-tazobactam. The highest resistance rate was to tetracycline (73.7%), followed by nitrofurantoin (53.3%) and ampicillin (50%). However, the difference between the proportion of chicken carcasses containing nitrofurantoin susceptible and resistant isolates was not statistically significant. Eight samples contained isolates that were resistant to ceftriaxone (26.7%), indicating ESBL producers. The *Salmonella* in all eight samples were also resistant to amoxicillin-clavulanic acid but susceptible to piperacillin-tazobactam and cefepime.

**Table 3.** Antibiotic Resistance Rates in *Salmonella* Obtained from Retail Chicken Carcasses in Qatar

Group	Drug	Percentage of chicken samples with resistant Salmonella (*n*)
Amphenicols	Chloramphenicol	3.3% (1)
Beta-lactams/penicillins	Ampicillin	50% (15)
	Amoxicillin/clavulanic acid	26.7% (8)
	Piperacillin/tazobactam	0.0% (0)
Cephalosporins (3rd gen.)	Ceftriaxone	26.7% (8)
Cephalosporins (4th gen.)	Cefepime	0.0% (0)
Carbapenems	Ertapenem	0.0% (0)
	Meropenem	0.0% (0)
Polymyxins	Colistin	6.7% (2)
Quinolones	Ciprofloxacin	23.3% (7)
Trimethoprims	Trimethoprim/sulfamethoxazole	6.7% (2)
Tetracyclines	Tetracycline	73.7% (22)
Other antibiotics	Fosfomycin	10% (3)
	Nitrofurantoin	53.3% (16)

**FIG. 3.**
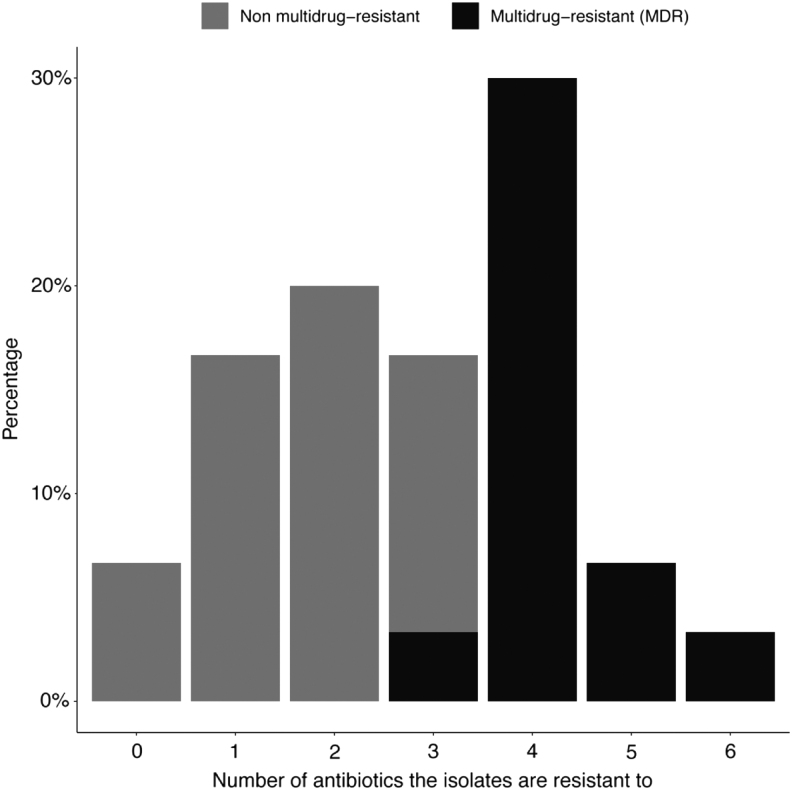
The distribution of the number of phenotypic resistances to up to 14 antibiotics among the *Salmonella* isolates (*n* = 30) from retail chicken carcasses in Qatar. Multidrug resistance is defined as the resistance to ≥3 antibiotic classes.

Two samples contained isolates resistant to colistin (6.7%); each was obtained from a different hypermarket. Incidentally, both isolates were resistant to ciprofloxacin, tetracycline, and nitrofurantoin. However, they were susceptible to β-lactams. Approximately 93.3% of the samples contained isolates that were resistant to at least one antibiotic; 16.67%, 20%, 16.67%, 30%, 6.67%, and 3.33% contained isolates that were resistant to 1, 2, 3, 4, 5, and 6 antibiotics, respectively (Fig. 3). Thirteen chicken samples (43.33%) contained MDR *Salmonella*, defined as resistant to ≥3 antibiotic classes. A Goodman-Kruskal τ was measured for each pair of phenotypic resistances to assess the pair-wise associations (Supplementary Fig. S2). An association was observed between ceftriaxone and amoxicillin-clavulanic acid resistance (τ = 1).

### Phenotypic resistance profile of *Salmonella* by storage temperature, hypermarket chain, and source

There was a significant difference between the proportions of resistance to amoxicillin-clavulanic acid and ceftriaxone between chilled and frozen chicken (*p* = 5 × 10^−4^) in addition to local and imported chicken (*p* = 4.7 × 10^−5^). All the isolates resistant to amoxicillin-clavulanic acid were also resistant to ceftriaxone (isolates aR127, aR215, aR217, aR218, cR104, cR231, cR232, and bD34). Additionally, all of these isolates were obtained from imported frozen chicken. No significant difference was found between the other antibiotics and storage temperature or source (Supplementary Fig. S3). Moreover, no significant difference was observed between the three hypermarket chains. As for MDR *Salmonella*, there was a significant difference between chilled and frozen chicken, with MDR being more likely in frozen chicken (OR = 16.88, 95% CI: 2.55–111.47, *p* = 0.002). No significant difference was observed between local and imported chicken and the hypermarkets.

## Discussion

Antimicrobial overuse and misuse are the main contributing drivers in the development and the global spread of AMR.^16^ Many of the antimicrobials used to treat infections in humans are also used for treatment and prophylaxis in farm animals.^16^ Antimicrobial overuse may increase AMR in livestock, creating a farm to fork transmission path. Studies that compared AMR pathogens in animals, food, and humans found significant similarities in AMR genes and plasmids and, to a lesser extent, between the pathogens, indicating a route from animal to human through food (particularly poultry).^18–21^ Thus, adopting a one health approach in AMR surveillance is necessary. While various studies have been conducted to investigate the epidemiology of AMR in the human population in Qatar, data on livestock, food, and the environment is still limited.

Antimicrobial-resistant *Salmonella* is classified as a serious threat by the Centers for Disease Control (CDC) in the United States. Fluoroquinolone-resistant *Salmonella* is classified as a high-priority pathogen by the World Health Organization (WHO).^31,32^ Additionally, the main route for *Salmonella* infections is through contaminated food, particularly poultry products. To that extent, this study aimed to investigate the prevalence of *Salmonella* in retail chicken carcasses and their AMR profiles. We report on the prevalence, and AMR profiles of *Salmonella* isolates from retail chicken carcasses. These chicken samples were obtained from three hypermarket chains across the two most population-dense municipalities, Doha and Al-Rayyan.

The prevalence of *Salmonella* in retail chicken varies widely across the globe, ranging from as low as 2.7% in Brazil to 97.9% in Myanmar, with most recent reports in the range 25%–55%.^33–41^ In the present study, the prevalence of *Salmonella* was found to be 11.11%. No significant difference in the prevalence of *Salmonella* was found between the two municipalities or the three hypermarket chains. In contrast, the prevalence was significantly higher in local compared to imported chicken (OR = 2.56, 95% CI: 1.18–5.53, *p* = 0.016). All the local chicken in the study was chilled; however, no significant difference was found between the storage temperatures (chilled vs. frozen). This finding is in contrast to other studies that found a significantly higher prevalence in chilled chicken.^36,42^

The higher prevalence in chilled chicken in those studies could be attributed to the fluctuating storage temperatures associated with chilled chicken transport. The relation with temperature fluctuation is further supported by the higher prevalence of *Salmonella* in retail chicken in summer than winter, likely due to the considerable temperature difference between the environment and storage conditions.^37^ Another reason is that thawing frozen chicken may reduce the viability of *Salmonella*.^36^ Additionally, multiple studies found an association between the type of chicken production company and *Salmonella* prevalence. A study in Colombia found that integrated companies (those that own and control all stages of production) have a lower prevalence in the final chicken product than non-integrated companies.^36^ The difference is likely related to fluctuations in temperature during transport between the various involved entities and differences in quality and safety standards.

Twenty-eight of the 30 *Salmonella* contaminated retail chicken carcasses in this study (93.3%) had isolates resistant to at least one antibiotic. The highest resistance rate observed was to tetracycline (73.3%), commonly used in animal feed as prophylaxis and a growth promoter.^16^ The high rate of tetracycline-resistance is consistent with other reports that found an increase in resistance rates over the past few years.^43^ Other frequently overused antibiotics in animal feed and veterinary medicine include fluoroquinolones, cephalosporins, and colistin.^16^ Overuse of these antibiotics in animals is a key driver in the increase and spread of AMR. Of those antibiotics, fluoroquinolones, ampicillin, and amoxicillin-clavulanic acid are first-line drugs in treating salmonellosis.^43^

In this study, the resistance rates for the three antibiotics in contaminated chicken were 50%, 26.7%, and 23.3% for ampicillin, amoxicillin-clavulanic acid, and ciprofloxacin, respectively. The resistance rates for these antibiotics vary between regions.^37,39,40^ All the amoxicillin-clavulanic acid-resistant isolates are likely ESBL-producers, as indicated by their resistance to the third generation cephalosporin ceftriaxone. Notably, all eight ESBL-producers were isolated from imported frozen chicken. Of greater concern is that all the potential ESBL-producers, and the majority of ciprofloxacin-resistant isolates, are MDR (Supplementary Table S1). Thirteen chicken carcasses were contaminated with MDR *Salmonella* (43.3%). The MDR prevalence was significantly higher in frozen chicken (OR = 16.88, 95% CI: 2.55–111.47, *p* = 0.002). The prevalence of MDR found in our study is similar to a study performed in Brazil, which found that 53.2% of the *Salmonella* isolated from retail chicken were MDR.^35^ However, the same study also reported a 2.7% prevalence of *Salmonella*.

Nevertheless, several studies in Europe reported high rates of MDR *Salmonella* in chicken imported from Brazil, particularly serovar Heidelberg.^44–46^ These studies suggest that the food hygiene regulations need to be revised to curb the spread of MDR *Salmonella*. A more detailed genomic investigation is necessary to characterize the isolates better and elucidate whether a similar trend is occurring in Qatar.

## Conclusions

Our findings revealed a moderate prevalence of *Salmonella* in retail chicken carcasses in Qatar, with 11.1% of retail chicken carcasses contaminated with *Salmonella*. However, the high rate of MDR (43.3% of the *Salmonella* isolates) and resistance to first-line drugs are of significant concern. These isolates can potentially be transmitted along the food chain to humans and eventually back into the environment and other animals.^16^ This highlights the importance of incorporating antimicrobial susceptibility testing in food hygiene monitoring. Interestingly, the locally produced chicken was associated with a higher prevalence of *Salmonella*. In contrast, the imported chicken was associated with a higher rate of MDR *Salmonella*. The association between imported chicken and MDR indicates a possible route of the global dissemination of AMR that emphasizes the need for an interdisciplinary and multicountry effort to tackle the issue on a global scale.

## Supplementary Material

Supplemental data

Supplemental data

Supplemental data

Supplemental data
