# Supplementary material for: Retail Chicken Carcasses as a Reservoir of Multidrug-Resistant Salmonella
Source: Microb Drug Resist. 2022 Jul 13;28(7):824–31. doi: 10.1089/mdr.2021.0414 (PMC9347385; doi:10.1089/mdr.2021.0414)
Supplement: Supplemental data [file Supp_Fig1.docx]

*
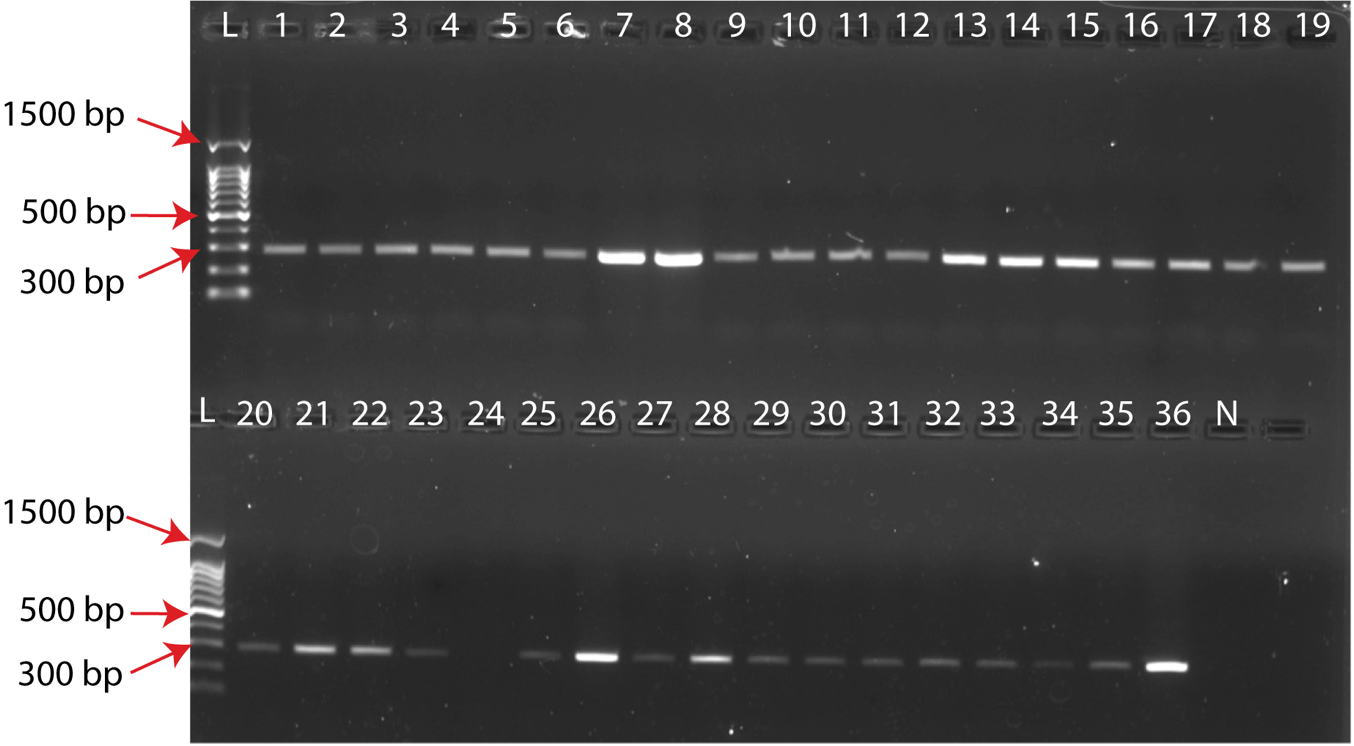
*

**Figure S1.** Detection of the invA gene PCR product in suspected *Salmonella* isolates recovered from retail chicken carcasses via agarose gel electrophoresis. L: GelPilot 100 bp Plus Ladder (Qiagen, Germany). N: Negative Control, nuclease free water. Lanes 1-23 and 25-26 invA positive isolates. Lane 24 invA negative isolate.
