# Supplementary material for: Retail Chicken Carcasses as a Reservoir of Multidrug-Resistant Salmonella
Source: Microb Drug Resist. 2022 Jul 13;28(7):824–31. doi: 10.1089/mdr.2021.0414 (PMC9347385; doi:10.1089/mdr.2021.0414)
Supplement: Supplemental data [file Supp_Table1.docx]

**Table S1.** The *Salmonella* contaminated samples included in this study, their sources and storage temperature, and antibiotic susceptibility profiles

| Sample_id | | Date | Location | Hypermarket_chain | Storage_temp | Local_import | AMP | AMC | TZP | CRO | FEP | ETP | MEM | CIP | CHL | TE | SXT | CST | FOF |
| --- | --- | --- | --- | --- | --- | --- | --- | --- | --- | --- | --- | --- | --- | --- | --- | --- | --- | --- | --- |
| aD2 | 19-Nov-17 | | Doha | A | chilled | local | R | S | S | S | S | S | S | I | S | R | S | S | S |
| aR15 | 25-Nov-17 | | Al-Rayyan | A | chilled | local | I | S | S | S | S | S | S | R | S | R | S | R | S |
| aD27 | 2-Nov-17 | | Doha | A | chilled | local | I | S | S | S | S | S | S | I | S | R | S | S | S |
| aD28 | 2-Nov-17 | | Doha | A | chilled | local | R | S | S | S | S | S | S | R | I | R | S | S | S |
| aR64 | 6-Dec-18 | | Al-Rayyan | A | chilled | local | S | S | S | S | S | S | S | I | S | S | S | S | S |
| aD81 | 30-Dec-17 | | Doha | A | frozen | imported | R | S | S | S | S | S | S | S | S | S | S | S | R |
| aR101 | 6-Jan-18 | | Al-Rayyan | A | frozen | imported | S | S | S | S | S | S | S | I | S | S | S | S | S |
| aR127 | 4-Feb-18 | | Al-Rayyan | A | frozen | imported | R | R | S | R | S | S | S | I | S | R | S | S | S |
| aR215 | 3-Nov-18 | | Al-Rayyan | A | frozen | imported | R | R | S | R | I | S | S | I | S | R | S | S | S |
| aR217 | 3-Nov-18 | | Al-Rayyan | A | frozen | imported | R | R | S | R | S | S | S | I | S | R | S | S | S |
| aR218 | 3-Nov-18 | | Al-Rayyan | A | frozen | imported | R | R | S | R | S | S | S | I | S | R | S | S | S |
| aD241 | 4-Jan-18 | | Doha | A | chilled | imported | S | S | S | S | S | S | S | I | S | R | S | S | S |
| cR22 | 25-Nov-17 | | Al-Rayyan | B | chilled | local | S | S | S | S | S | S | S | I | S | S | S | S | R |
| cR46 | 9-Nov-17 | | Al-Rayyan | B | chilled | local | R | S | S | S | S | S | S | I | S | R | S | S | S |
| cR47 | 9-Nov-17 | | Al-Rayyan | B | chilled | local | S | S | S | S | S | S | S | I | S | R | S | S | S |
| cD61 | 19-Dec-17 | | Doha | B | chilled | local | R | S | S | S | S | S | S | I | S | R | R | S | S |
| cD62 | 20-Dec-17 | | Doha | B | chilled | local | R | S | S | S | S | S | S | I | S | R | S | S | S |
| cR104 | 6-Jan-18 | | Al-Rayyan | B | frozen | imported | R | R | S | R | S | S | S | I | S | R | S | S | S |
| cR204 | 3-Nov-18 | | Al-Rayyan | B | chilled | local | S | S | S | S | S | S | S | I | S | S | S | S | S |
| cR210 | 3-Nov-18 | | Al-Rayyan | B | chilled | local | S | S | S | S | S | S | S | R | S | R | R | R | R |
| cR231 | 3-Nov-18 | | Al-Rayyan | B | frozen | imported | R | R | S | R | S | S | S | I | S | R | S | S | S |
| cR232 | 3-Nov-18 | | Al-Rayyan | B | frozen | imported | R | R | S | R | S | S | S | R | S | R | S | S | S |
| bD34 | 2-Nov-17 | | Doha | C | frozen | imported | R | R | S | R | S | S | S | R | S | R | I | S | S |
| bD111 | 14-Jan-18 | | Doha | C | chilled | local | S | S | S | S | S | S | S | I | I | R | I | S | S |
| bD112 | 14-Jan-18 | | Doha | C | chilled | local | S | S | S | S | S | S | S | I | I | R | S | S | S |
| bD113 | 14-Jan-18 | | Doha | C | chilled | local | I | S | S | S | S | S | S | R | S | R | S | S | S |
| bD114 | 14-Jan-18 | | Doha | C | chilled | local | S | S | S | S | S | S | S | I | S | S | S | S | S |
| bD150 | 4-Mar-18 | | Doha | C | chilled | imported | S | S | S | S | S | S | S | I | S | S | S | S | S |
| bD163 | 4-Mar-18 | | Doha | C | frozen | imported | R | S | S | S | S | S | S | R | S | R | S | S | S |
| bD268 | 4-Feb-18 | | Al-Rayyan | C | chilled | imported | S | I | S | S | S | S | S | I | R | S | S | S | S |
