# Supplementary material for: Retail Chicken Carcasses as a Reservoir of Multidrug-Resistant Salmonella
Source: Microb Drug Resist. 2022 Jul 13;28(7):824–31. doi: 10.1089/mdr.2021.0414 (PMC9347385; doi:10.1089/mdr.2021.0414)
Supplement: Supplemental data [file Supp_Fig2.docx]

*
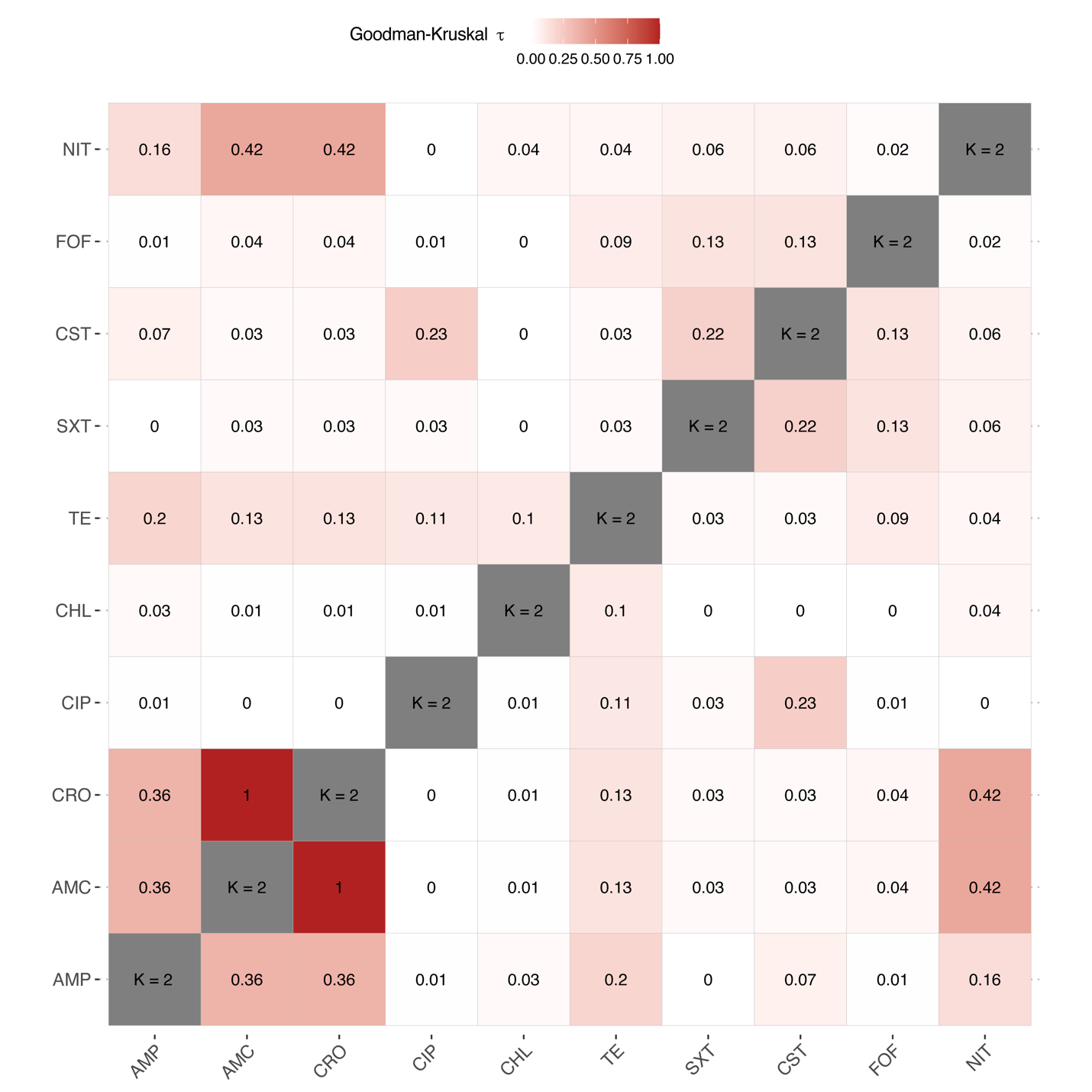
*

**Figure S2.** Goodman-Kruskal τ measures of associations between the phenotypic resistance to various antibiotics in *Salmonella* isolated from retail chicken carcasses in Qatar. K: number of distinct values. AMC: amoxicillin-clavulanic acid, AMP: ampicillin, CHL: chloramphenicol, CIP: ciprofloxacin, CRO: ceftriaxone, CST: colistin, FOF: Fosfomycin, NIT: nitrofurantoin, SXT: sulfamethoxazole-trimethoprim, TE: tetracycline. Goodman-Kurskal τ is an asymmetric measure of association that ranges between 0 (no association) to 1 (complete association). All τ measures were bidirectional. The results indicate that there is an association between the resistance to ceftriaxone (CRO) and amoxicillin-clavulanic acid (AMC).
