# Supplementary material for: Retail Chicken Carcasses as a Reservoir of Multidrug-Resistant Salmonella
Source: Microb Drug Resist. 2022 Jul 13;28(7):824–31. doi: 10.1089/mdr.2021.0414 (PMC9347385; doi:10.1089/mdr.2021.0414)
Supplement: Supplemental data [file Supp_Fig3.docx]

**

**Figure S3.** The percentage of retail chicken carcasses carrying antimicrobial-resistant *Salmonella* stratified by source (local vs. imported) and storage temperature. The bars show the percentage of resistant *Salmonella* in each category out of the total resistant ones for the antibiotic. AMC: amoxicillin-clavulanic acid, CHL: chloramphenicol, CIP: ciprofloxacin, CRO: ceftriaxone, CST: colistin, FOF: Fosfomycin, SXT: sulfamethoxazole-trimethoprim, TE: tetracycline. The number of resistant isolates is shown above the bars*.* ****: P < 0.0001.
